# Interplay of Psychosocial, Clinical and Modulation Mechanisms in Chronic TMD‐Related Disability

**DOI:** 10.1111/joor.70008

**Published:** 2025-06-26

**Authors:** Rafaela Stocker Salbego, Paulo César Rodrigues Conti, Matheus Herreira‐Ferreira, Beatriz Amaral de Lima Netto, Tiffany Karoline Barroso Santos, Yuri Martins Costa, Leonardo Rigoldi Bonjardim

**Affiliations:** ^1^ Department of Biological Sciences, Bauru School of Dentistry University of São Paulo Bauru Brazil; ^2^ Department of Prosthodontics and Periodontology, Bauru School of Dentistry University of São Paulo Bauru Brazil; ^3^ Department of Biosciences, Piracicaba School of Dentistry University of Campinas Piracicaba Brazil

**Keywords:** chronic pain, orofacial pain, temporomandibular joint disorders

## Abstract

**Background:**

Temporomandibular disorder (TMD) is a multifactorial condition characterised by pain and functional impairment in the orofacial region. Although psychosocial and psychophysical factors significantly influence chronic pain, their combined impact on functional disability remains poorly understood.

**Objectives:**

To investigate the influence of clinical, psychosocial and psychophysical variables on functional disability related to chronic pain in muscular TMD, utilising principal component analysis (PCA) and logistic regression.

**Methods:**

A cross‐sectional study with 77 women with painful TMD assessed clinical parameters (pain intensity, frequency, comorbidities and duration), psychosocial characteristics (stress, anxiety, depression, sleep quality and pain catastrophising) and psychophysical assessments (pressure pain threshold, mechanical pain threshold and conditioned pain modulation). PCA identified principal components, which were analysed using logistic regression to evaluate their association with chronic pain grades through the Graded Chronic Pain Scale.

**Results:**

Five principal components were identified: psychosocial, clinical pain, duration and extent of pain, pain modulation and sensory. The clinical pain component significantly predicted functional disability across all chronic pain grades (*p* < 0.05). The psychosocial component was a strong predictor of higher disability levels, whereas the pain modulation component showed a protective effect at the highest disability grade.

**Conclusion:**

Functional disability in TMD is influenced by a complex interplay of clinical, psychosocial and psychophysical factors. Among five components founded, clinical pain and psychosocial components emerged as the most significant predictors of functional disability, with pain modulation providing a protective role at higher disability levels. This study elucidates the intricate interplay between clinical, psychosocial and psychophysical factors contributing to functional disability in chronic TMD‐related pain.

## Graphical abstract

Principal components associated with chronic TMD‐related disability. A multivariate model using principal component analysis and logistic regression identified five key components—psychosocial, clinical pain, duration and extent of pain, pain modulation, and sensory function—that interact to influence functional disability in individuals with muscular temporomandibular disorder (TMD).

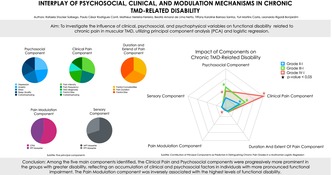

## 1 Introduction

Temporomandibular disorder (TMD) is a complex and multifactorial condition characterised by pain and functional impairment in the orofacial region, often involving the masticatory muscles, temporomandibular joints and associated structures [1, 2]. Studies have shown that individuals with TMD, particularly those experiencing chronic muscular pain, exhibit a combination of clinical, psychosocial and psychophysical factors that can amplify pain perception and exacerbate functional disability [3, 4].

To better understand the variability and complexity of TMD, the use of standardised tools such as the Graded Chronic Pain Scale (GCPS) is crucial. The GCPS facilitates the assessment of both pain intensity and its functional impact, categorising chronic pain into different grades. This approach enables the identification of patients with greater psychosocial and functional impairment, allowing for more personalised interventions [5, 6].

Recent evidence highlights the critical role of psychosocial factors—such as anxiety, depression, pain catastrophising and sleep quality—in chronic pain states. The interaction between these factors and clinical pain components, such as frequency and intensity, appears to play a significant role in the exacerbation of TMD symptoms [7, 8, 9]. Additionally, psychophysical variables, assessed through quantitative sensory testing (QST) and conditioned pain modulation (CPM), provide objective measures of pain sensitivity and modulation, aiding in the identification of specific sensory profiles in patients with chronic pain [10, 11, 12].

Studies employing the GCPS in TMD research have been instrumental in elucidating how pain intensity and functional impairment influence the pain experience across different levels of severity. The literature suggests that high GCPS scores are associated with increased psychosocial symptoms and a more pronounced impact on quality of life in TMD patients [13]. However, most studies to date have examined these factors in isolation, without delving deeply into the interactions among clinical, psychosocial and psychophysical variables [14, 15, 16, 17]. Furthermore, few studies have utilised multivariate analyses to explore how these domains collectively contribute to the chronic pain grades defined by the GCPS. This gap limits the understanding of TMD as a multidimensional and interrelated condition, where psychosocial factors and pain modulation mechanisms interact to shape pain perception and functional impairment.

This study aims to address this gap by investigating whether clinical, psychosocial and psychophysical variables influence the degree of disability associated with chronic pain in patients with muscular TMD. We employ principal component analysis (PCA) to identify underlying patterns within complex datasets, combined with multinomial logistic regression. Our hypothesis is that PCA will reveal patterns contributing incrementally to higher degrees of disability associated with chronic pain. This approach has the potential to enhance patient phenotyping, offering a more comprehensive understanding of functional impairment in TMD.

## 2 Materials and Methods

### 2.1 Study Design

This cross‐sectional study was submitted to and approved by the Research Ethics Committee of the Bauru School of Dentistry at the University of São Paulo (CAAE 82201818.3.0000.5417). It was conducted in accordance with the Helsinki Declaration and followed the STROBE (The Strengthening the Reporting of Observational Studies in Epidemiology) [18] guidelines for cross‐sectional studies. All individuals who agreed to participate in the primary study signed an informed consent form.

This cross‐sectional study represents a secondary analysis of data derived from 77 patients diagnosed with painful TMD (myalgia and/or headache attributed to TMD) according to the Diagnostic Criteria for Temporomandibular Disorders (DC/TMD) with pain persisting for at least 90 days; additionally, the severity of chronic pain was assessed using the GCPS [19, 20]. All participants with TMD presented with myalgia in the masseter muscle and could also have myalgia in the temporal muscle and/or an associated diagnosis of arthralgia. Recruitment occurred between October 2018 and December 2019. The exclusive selection of the female gender aimed to mitigate the influence of sex, which may impact pain thresholds [21, 22]. Inclusion criteria encompassed (a) age over 18 years; (b) diagnosis of myalgia and/or headache attributed to TMD either associated or not with other painful diagnoses of TMD, that is, arthralgia; and (c) pain persisting for at least 90 days. Exclusion criteria comprised (a) men; (b) uncontrolled systemic disorders, such as diabetes, hypertension or endocrine disorders; (c) congenital or developmental changes, for example, aplasia, hyperplasia, dysplasia or neoplasms; (d) presence of neuropathic pain; (e) treatment with serotonin–norepinephrine reuptake inhibitors (SNRIs) in the past 12 months; (f) pregnancy or lactation; and (g) treatment for TMD in the past 3 months.

All evaluations were conducted by the same examiner, a specialist in temporomandibular disorders and orofacial pain. All included individuals underwent clinical, psychosocial and psychophysical assessments as detailed below. The questionnaires used were administered in their translated and validated Brazilian Portuguese versions [23, 24, 25, 26, 27].

### 2.2 Assessed Variables

#### 2.2.1 Clinical Parameters

In this study, participants were instructed to complete an anamnesis form, which included reporting the intensity of pain experienced in the past week using a visual analogue scale ranging from 0 to 10, the duration of pain since its onset (in months) and the frequency of pain days during the past week. The number of painful comorbidities was recorded based on patient‐reported medical diagnoses of such conditions. Additionally, variables related to the number of painful sites across the body and the GCPS were collected, as detailed below. After the TMD diagnosis was established, the total number of TMD diagnoses for each patient was also documented.

##### 2.2.1.1 Number of Painful Sites

The number of painful sites across the body was assessed using the evaluation tool from the DC/TMD [19]. Participants were instructed to mark on a ‘pain drawing’ the areas where they experienced pain in the past month. The examiner then counted the marked areas, which could range from 0 to 45 sites. Higher values indicated a greater spread of pain throughout the body [19, 20].

##### 2.2.1.2 Graded Chronic Pain Scale

###### 2.2.1.2.1 Pain Intensity

The GCPS was used to assess the average pain intensity over the past month (chronic pain intensity—CPI). This was calculated as the arithmetic mean of three scales, multiplied by 10, where 0 represents ‘no pain’ and 10 represents ‘the worst possible pain’. The scales evaluated pain at the moment, the worst pain and the average pain experienced in the past 30 days [19, 20].

###### 2.2.1.2.2 Functional Disability

Functional disability was measured using a 0–6 point scale. This score was calculated by taking the arithmetic mean of three scales, multiplied by 10, which assessed the severity of interference in daily activities, work and social activities. The interference ranged from 0 (‘no interference’) to 10 (‘unable to perform any activity’). Additional points were added based on the number of days participants reported being unable to perform activities due to pain in the past month [19, 20].

###### 2.2.1.2.3 Pain Grading

The GCPS categorises pain into the following grades:

– Grade 0: No pain in the past 6 months.
– Grade I: Pain intensity below 50 points and less than 3 points of functional disability.
– Grade II: Pain intensity above 50 points and less than 3 points of functional disability.
– Grade III: Functional disability between 3 and 4 points, regardless of pain intensity.
– Grade IV: Functional disability between 5 and 6 points, regardless of pain intensity.

#### 2.2.2 Psychosocial and Sleep Quality Characterisation

Psychosocial characteristics, such as anxiety level, depression, sleep quality, degree of pain‐related catastrophising and stress level, were assessed using the following questionnaires: Hospital Anxiety and Depression Scale (HADS), Pittsburgh Sleep Quality Index (PSQI), Pain Catastrophising Scale (PCS) and Perceived Stress Scale (PSS).

##### 2.2.2.1 Hospital Anxiety and Depression Scale

This questionnaire is a self‐report instrument consisting of 14 multiple‐choice questions, divided into two alternating subscales: one for anxiety (7 questions) and the other for depression (7 questions). Scores range from 0 to 21 points; higher scores indicate more significant symptoms of anxiety and/or depression [28, 29].

##### 2.2.2.2 Pain Catastrophising Scale

This questionnaire assesses catastrophic thoughts about pain, with individuals indicating the frequency of catastrophic thoughts during intense pain experiences. It consists of 13 statements, rated on a frequency scale ranging from 0 to 5 (0 = almost never, 5 = almost always). The total score is calculated by summing all items, ranging from 0 to 52 points; higher scores reflect a higher degree of catastrophising about pain [24, 30].

##### 2.2.2.3 Perceived Stress Scale

This questionnaire measures the perceived stress level over the past month, considering the overall context of the individual's life. It comprises 14 items aimed at assessing how unpredictable, uncontrollable and overloaded the respondent perceives their life. The total scale score is the sum of the scores for these 14 items, ranging from 0 to 56; higher scores indicate greater perceived stress [25, 31].

##### 2.2.2.4 Pittsburgh Sleep Quality Index

This questionnaire aims to differentiate individuals who sleep ‘well’ from those who sleep ‘poorly’, providing clinical insights into various sleep disorders that can impact sleep quality. It comprises 19 self‐report questions evaluating multiple aspects related to sleep quality, including estimates of sleep duration and latency, and the frequency and severity of specific sleep disturbances. Higher scores indicate poorer sleep quality [23, 32].

#### 2.2.3 Psychophysical Characterisation

Pressure pain threshold (PPT), mechanical pain threshold (MPT) and conditioned pain modulation (CPM). All tests were conducted on the most painful point of the masseter muscle in each patient (determined during clinical examination).

##### 2.2.3.1 Pressure Pain Threshold

As measurements of the PPT were conducted using a digital dynamometer equipped with a flat circular tip of 1 cm^2^ (Medoc AlgoMed, Medoc Ltd. Advanced Medical Systems, Israel). This device applied a constant and gradually increasing pressure of approximately 0.5 kg/cm^2^/s. Prior to the examination, individuals were trained to press the button on the device when the pressure sensation turned into a slightly painful stimulus. Thus, this test measures the PPT, and the arithmetic mean of three consecutive measurements was considered as the threshold [33].

##### 2.2.3.2 Mechanical Pain Threshold

This test involves the use of Semmes‐Weinstein monofilaments to determine the MPT. The kit comprises 20 nylon Von Frey monofilaments (Touch‐Test Sensory Evaluators, North Coast Medical, Morgan Hill, CA, USA) of varying diameters calibrated to exert specific forces that increase with the filament gauge. The force applied by the monofilament ranges from 0.008 to 300 g/mm^2^. Each monofilament was applied perpendicularly to the area under assessment, exerting slight pressure until the filament bent. Participants were instructed to verbally report when they felt a sensation of ‘prick, pinprick or a slightly painful sting’ at the point of contact with the monofilaments. The tests commenced with the least calibrated filament (0.008 g/mm^2^) and proceeded sequentially with increasingly calibrated filaments until the participant verbally reported feeling a slightly painful prick/sting, as instructed at the beginning of the test. This was considered a positive stimulus (+). After this positive report, the order was reversed, and the next lower value filament was used until the participant no longer felt the application of the noxious tactile stimulus (touch, not a painful prick/sting). This was considered a negative stimulus (−). This measurement continued until obtaining five negative (descending) and five positive (ascending) stimuli, and the geometric mean of these repetitions was calculated [33].

##### 2.2.3.3 Conditioned Pain Modulation

Conditioned pain modulation involved a series of steps to assess pain modulation. Initially, a PPT test stimulus was applied using a pressure algometer equipped with a flat circular tip of 1 cm^2^, delivering a constant and gradually increasing pressure of approximately 0.5 kg/cm^2^/s. Before the examination, individuals were trained to press a button registering the force applied by the device when the pressure sensation turned into a slightly painful stimulus. This test measured the PPT, and the arithmetic mean of three consecutive measurements was considered the calculated threshold. Subsequently, the individual was instructed to immerse the contralateral hand (relative to the location of the PPT test) into a container filled with cold water between 10°C and 16°C, adjusted according to the individual's tolerance, inducing a pain intensity of 3 on a scale from 0 to 10. For the DTM group, this was the hand with less pain. This initial PPT was regarded as the initial test stimulus (ITS), whereas the hand immersion in cold water was considered the conditioning stimulus (CS). Following the CS, the PPT test was repeated, serving as the final test stimulus (FTS) [34, 35]. The evaluation protocol was sequential, meaning the FTS was immediately repeated after the application of the CS. Consequently, CPM was calculated as the absolute difference between the ‘ITS and FTS’ [36, 37, 38].

### 2.3 Statistical Analysis

Data analysis was conducted using Jamovi version 2.6.2 software (https://www.jamovi.org). Descriptive statistics were computed for the demographic and study variables, including frequencies and percentages (for categorical variables), means and standard deviations (for continuous variables) or medians with 25th and 75th percentiles (for non‐parametric variables). The normality of the data was assessed using the Shapiro–Wilk test (*p* < 0.05). To perform the PCA, Bartlett's test of sphericity (*p* < 0.001) and the Kaiser‐Meyer‐Olkin (KMO) measure of sampling adequacy were evaluated, with a global KMO value of 0.60 being considered acceptable. The varimax rotation method was applied, and a scree plot was used to identify components with eigenvalues greater than 1. Subsequently, multinomial logistic regression was conducted to evaluate the influence of principal components on the odds ratios (OR) of individuals belonging to specific levels of chronic pain grading compared to Grade I. The independence of observations was ensured through PCA, which generates independent components. Additionally, the variance inflation factor (VIF) was checked, with all dependent variables showing VIF values equal to 1. Partial residual plots were inspected visually to confirm the linearity of the logit. Effect sizes for each predictor in the multinomial logistic regression models were reported using OR with corresponding 95% confidence intervals. Additionally, the overall model fit was evaluated by reporting Nagelkerke's pseudo‐*R* ^2^.

In this analysis, chronic pain grades based on the GCPS (I, II, III and IV) served as the criterion variable, with all principal components included as predictors. Statistical significance was set at *p* < 0.05.

## 3 Results

Seventy‐seven women with chronic painful muscular TMD (DC/TMD) were cross‐sectionally evaluated for psychosocial and sleep variables (stress, anxiety, depression, pain catastrophising and sleep quality), psychophysical variables (PPT, MPT and CPM of the masseter muscle) and clinical variables (pain duration in months, pain frequency in the last week, number of painful comorbidities, number of painful sites, VAS score for the last week and number of TMD diagnoses). The GCPS was also assessed. The mean age of participants was 38 years (interquartile range: 34–50 years), with no significant differences in age across chronic pain grade groups (*p* = 0.789, *F* = 0.351, ANOVA). Descriptive data are presented as means and standard deviations (for normally distributed variables) or medians and interquartile ranges (for skewed data) in Tables 1, 2, 3.

**TABLE 1:** Descriptive analysis for psychosocial and sleep parameters.

GCPS	*N*	Depression mean (DP)	Anxiety mean (DP)	Stress mean (DP)	Sleep quality mean (DP)	Catastrophising mean (DP)
I	8	4.88 (3.94)	8.38 (4.78)	25.5 (10.8)	7.63 (3.58)	15.5 (12.3)
II	42	6.29 (3.12)	8.83 (3.83)	28.3 (9.48)	8.83 (3.78)	28.8 (11.5)
III	17	8.88 (3.67)	10.8 (3.71)	31.8 (7.70)	9.35 (3.90)	33.8 (10.4)
IV	10	8.30 (3.89)	9.80 (3.85)	28.6 (10.7)	10.3 (4.22)	29.5 (12.7)

Abbreviations: GCPS, Graded Chronic Pain Scale; SD, standard deviation.

**TABLE 2:** Descriptive analysis for clinical parameters.

GCPS	N	Pain intensity	Pain frequency	TMD diagnoses	Painful sites	Painful comorbidities	Pain duration
I	8	5.50 (4.50–6.00)	4.50 (3.50–7.00)	2.50 (2.00–3.00)	4.50 (3.75–6.00)	0.50 (0.00–1.25)	21.0 (14.3–255)
II	42	7.00 (6.00–8.00)	7.00 (2.25–7.00)	3.00 (2.00–3.00)	6.00 (4.00–9.00)	1.00 (0.00–1.75)	48.0 (30.0–84.0)
III	17	8.00 (7.00–8.00)	4.00 (3.00–7.00)	3.00 (3.00–3.00)	4.00 (3.00–7.00)	1.00 (0.00–2.00)	108 (48.0–120)
IV	10	8.00 (6.63–8.75)	7.00 (3.50–7.00)	3.00 (3.00–3.00)	7.00 (4.00–10.8)	1.50 (1.00–2.75)	42.0 (15.0–60.0)

Abbreviations: GCPS, Graded Chronic Pain Scale; SD, standard deviation.

**TABLE 3:** Descriptive analysis for psychophysical parameters.

	GCPS	CPM	PPT	MPT
Mean	I	0.395	1.57	
	II	−0.0342	1.49	
	III	0.0130	1.58	
	IV	0.0453	1.16	
SD	I	0.322	0.641	
	II	0.441	0.720	
	III	0.575	1.07	
	IV	0.373	0.590	
Median	I			2.67
	II			1.89
	III			3.67
	IV			2.55
25–75 percentile	I			1.38–6.00
	II			0.777–9.82
	III			2.10–4.36
	IV			1.38–6.00

Abbreviations: CPM, conditioned pain modulation; GCPS, Graded Chronic Pain Scale; MPT, mechanical pain threshold; PPT, pressure pain threshold; SD, standard deviation.

To perform a PCA, Bartlett's test of sphericity was conducted (*χ* ^2^ = 239, df = 91, *p* < 0.001), along with the KMO measure of sampling adequacy, which yielded a global value of 0.604 using the varimax rotation method. Although individual KMO values for some specific variables were lower, the overall KMO value indicates that the analysis is viable. The number of components was determined based on eigenvalues greater than 1, and the scree plot revealed that five principal components met this criterion. The PCA (Table 4) identified five principal components with eigenvalues greater than 1: (1) Psychosocial component: This component encompassed psychosocial variables, including stress, anxiety, depression, pain catastrophising and sleep quality. (2) Clinical pain component: This component included variables such as pain frequency in the past week, pain intensity in the past week, the number of TMD diagnoses and the number of painful sites (with a relatively small loading). Additionally, pain catastrophising contributed to this component. (3) Duration and extent of pain component: This component comprised variables related to pain duration, the number of painful comorbidities and the number of painful sites. (4) Pain modulation component: This component included the PPT and CPM of the masseter. (5) Sensory component: This component included PPT and MPT of the masseter.

**TABLE 4:** Principal component analysis.

	Component					Singularity
	1	2	3	4	5	
Depression	0.881					0.219
Anxiety	0.819					0.294
Stress	0.806					0.270
Sleep quality	0.666					0.537
Catastrophysing	0.533	0.410			−0.305	0.446
Pain intensity		0.811				0.316
Pain frequency		0.612				0.506
TMD diagnoses		0.554				0.593
Painful sites		0.317	0.776			0.279
Painful comorbidities			0.672			0.501
Pain duration			0.672			0.488
CPM				0.818		0.243
PPT masseter				0.751	0.385	0.282
MPT masseter					0.903	0.160

*Note:* ‘Varimax’ rotation was used. This table displays the component loadings obtained through principal component analysis (PCA) with varimax rotation. The five identified components (1–5) are: psychosocial, clinical pain, pain duration and extent, pain modulation and sensory. Each row represents a variable included in the analysis, with the values indicating factor loadings (the correlation of the variable with the component). Higher values indicate a stronger contribution of the variable to the specific component. The ‘Singularity’ column shows the uniqueness of each variable, reflecting the proportion of variance not explained by the principal components.

In the multinomial logistic regression analysis (Tables 5 and 6), the five principal components were included as independent variables, while chronic pain grade (I, II, III and IV) served as the dependent variable, with group sizes of 8, 42, 17 and 10 individuals, respectively. The analysis yielded a Nagelkerke pseudo‐*R*‐squared value of 0.335. The global model test demonstrated statistical significance, with a chi‐square value of 30.3 (*p* = 0.011).

**TABLE 5:** Multinomial logistic regression model fit measures.

Model	Deviance	AIC	$R_{\mathrm{CS}}^{2}$	Global model test		
				*χ* ^2^	gl	*p*
1	148	184	0.335	30.3	15	0.011
**Omnibus likelihood ratio test**						
**Predictor**				***χ*** ^**2**^	**gl**	***p***
Psychosocial component				10.012	3	0.018
Clinical pain component				13.873	3	0.003
Duration and extent of pain component				1.256	3	0.740
Pain modulation component				4.580	3	0.205
Sensory component				0.330	3	0.954

*Note:* This table presents the model fit statistics and the contribution of principal components as predictors in distinguishing chronic pain grades. Global model test evaluates the overall fit of the model, reporting deviance, Akaike information criterion (AIC), Nagelkerke pseudo‐*R* ^2^ ($R_{\mathrm{CS}}^{2}$), chi‐square (*χ* ^2^), degrees of freedom (gl) and *p* values. Omnibus likelihood ratio test shows the individual contribution of each principal component to the model, with chi‐square (*χ* ^2^), degrees of freedom (gl) and *p* values for each predictor.

**TABLE 6:** Model coefficients.

Grade	Predictor	Estimatives	Standard error	*Z*	*p*	Odds ratio	Confidence interval (95%)	
							Lower limit	Upper limit
II–I	Intercept	2.70771	0.688	3.93605	< 0.001	14.995	3.8939	57.744
	Psychosocial component	0.89650	0.546	1.64320	0.100	2.451	0.8413	7.141
	**Clinical pain component**	**1.18103**	**0.524**	**2.25379**	**0.024**	**3.258**	**1.1665**	**9.098**
	Duration and extent of pain component	−0.32989	0.550	−0.59998	0.549	0.719	0.2447	2.112
	Pain modulation component	−0.79759	0.502	−1.58837	0.112	0.450	0.1683	1.205
	Sensory component	−0.00218	0.655	−0.00333	0.997	0.998	0.2763	3.604
III–I	Intercept	1.67652	0.724	2.31675	0.021	5.347	1.2946	22.084
	**Psychosocial component**	**1.58126**	**0.617**	**2.56250**	**0.010**	**4.861**	**1.4504**	**16.293**
	**Clinical pain component**	**1.52364**	**0.629**	**2.42120**	**0.015**	**4.589**	**1.3368**	**15.753**
	Duration and extent of pain component	−0.08933	0.595	−0.15002	0.881	0.915	0.2847	2.938
	Pain modulation component	−0.89820	0.563	−1.59529	0.111	0.407	0.1351	1.228
	Sensory component	−0.16455	0.701	−0.23486	0.814	0.848	0.2149	3.349
IV–I	Intercept	0.88912	0.802	1.10816	0.268	2.433	0.5049	11.725
	**Psychosocial component**	**1.37684**	**0.665**	**2.06900**	**0.039**	**3.962**	**1.0753**	**14.601**
	**Clinical pain component**	**2.19717**	**0.757**	**2.90132**	**0.004**	**9.000**	**2.0399**	**39.704**
	Duration and extent of pain component	−0.00295	0.618	−0.00477	0.996	0.997	0.2968	3.349
	**Pain modulation component**	**−1.32752**	**0.654**	**−2.03083**	**0.042**	**0.265**	**0.0736**	**0.955**
	Sensory component	−0.10510	0.731	−0.14383	0.886	0.900	0.2150	3.770

*Note:* This table displays the regression coefficients, standard errors, odds ratios and confidence intervals for the multinomial logistic regression comparing chronic pain grades. Predictors lists the principal components used as independent variables in the model. Odds ratios (OR) represents the likelihood of association between each predictor and transitions across chronic pain grades (e.g., Grade II vs. Grade I). Confidence intervals (95%) indicates the precision of the odds ratio estimates. Comparisons includes Grade II versus I, Grade III versus I and Grade IV versus I, highlighting the predictors' contributions across different levels of pain severity. The analysis examines the relationships between psychosocial, clinical and psychophysical components and the severity of functional disability due to chronic pain. Bolded values in the figure indicate statistically significant associations between specific components and the likelihood of higher disability grades.

The independence of observations was ensured through the PCA, as the derived components are mutually independent. Additionally, the VIF was assessed, showing values of 1 for all independent variables, indicating no multicollinearity. Partial residual plots were visually inspected to confirm the linearity of the logit, further validating the model's assumptions.

The model's results indicate that the clinical pain component stands out as a significant predictor across all levels. In the comparison between Grades II and I, the clinical pain component demonstrated an OR of 3.258 (95% CI: 1.1665–9.098, *p* = 0.024), suggesting an increased likelihood of belonging to a higher grade. In the comparison between Grades III and I, both the psychosocial (OR = 4.861, 95% CI: 1.4504–16.293, *p* = 0.010) and clinical pain components (OR = 4.589, 95% CI: 1.3368–15.753, *p* = 0.015) were significant, with the psychosocial component exhibiting a more pronounced impact. Finally, in the comparison between Grades IV and I, the clinical pain component remained highly significant (OR = 9.000, 95% CI: 2.0399–39.704, *p* = 0.004), while the psychosocial component (OR = 3.962, 95% CI: 1.0753–14.601, *p* = 0.039) and pain modulation (OR = 0.265, 95% CI: 0.0736–0.955, *p* = 0.042) also showed significant relevance.

## 4 Discussion

The main findings of this study indicate that disability associated with chronic pain in patients with muscle‐related TMD is influenced by clinical, psychosocial and psychophysical components on an ascending scale. Through the identification of five principal components—psychosocial, clinical pain, pain duration and extent, pain modulation and sensory components—this analysis underscores the intricate interplay of clinical, psychosocial and psychophysical factors in shaping disability severity. Notably, only the clinical pain, psychosocial and pain modulation components emerged as significant predictors of functional disability progression, highlighting the central role of these factors in chronic pain mechanisms.

The clinical pain component, which includes variables such as pain frequency, intensity, number of TMD diagnoses and number of painful sites, was consistently significant across all levels of chronic pain‐related disability. Its increasing impact at higher disability levels reinforces the interpretation that greater cumulative clinical burden is associated with more severe functional impairment. Previous studies have also highlighted that higher frequencies of pain episodes and greater reported pain intensity are associated with worse functional perception and quality of life, particularly in patients with chronic conditions such as TMD [14, 39, 40]. While pain intensity alone may not determine more severe functional disability levels, higher scores in the clinical pain component accompany such disability. This underscores the importance of comprehensive and continuous clinical evaluation, as the component reflects an accumulation of clinical factors—such as pain frequency, intensity and distribution—that exacerbate functional impairments.

The psychosocial component, encompassing stress, anxiety, depression, sleep quality and catastrophising, demonstrated a profound influence, particularly at higher levels of disability. The observed cumulative effect of these variables is consistent with existing evidence that psychosocial distress amplifies pain perception and disability [8, 9, 41]. This interaction demonstrates an interrelationship between these variables, such that an increase in one is likely accompanied by an increase in the others. This creates a cumulative effect of psychosocial variables as factors that intensify the pain experience in patients with TMD. These factors appear to act as amplifiers of the pain process, where heightened central nervous system responses to pain are influenced by emotional states, leading to greater pain perception [8].

The pain modulation component acts as a protective factor at level IV, the stage of highest complexity and severity of chronic pain, compared to level I. This component has been identified as a critical factor in the pain experience of chronic pain patients [42, 43]. Studies have shown that individuals with greater modulation capacity exhibit reduced responses to chronic pain, suggesting that effective modulation is protective against the development of more disabling pain [12, 38, 42, 44, 45, 46]. The present research reinforces these findings, suggesting that interventions aimed at improving pain modulation may benefit TMD patients by reducing the intensity and impact of chronic pain, particularly in those with more severe conditions. Thus, it becomes evident that the complexity of chronic pain assessment through the GCPS involves multiple interrelated dimensions, such as greater clinical and psychosocial impacts, and at level IV, the influence of pain modulation.

In contrast, the pain duration and extent component and the sensory component did not significantly impact the likelihood of functional disability progression. This finding underscores the complex nature of chronic pain, suggesting that clinical and emotional factors take precedence over sensory and temporal aspects in determining functional outcomes. The sensory component, in particular, appears to reflect a downstream effect of chronic pain rather than a determinant of its severity. Longitudinal studies would be valuable in further elucidating these dynamics, as they may clarify whether sensory profiles and pain duration contribute indirectly to functional outcomes through their interactions with psychosocial and clinical factors.

This study aligns with the concept that patients with higher levels of chronic pain‐related functional disability exhibit greater complexity, characterised by multiple interacting factors. PCA identified distinct patterns within these variables, and the clustering of psychosocial and clinical factors suggests potential interdependence. For instance, high loadings on both the psychosocial and clinical pain components suggest that changes in emotional distress may co‐occur with shifts in pain perception and frequency. The significant portion of total variance explained by both components suggests they are capturing different facets of the same complex issue—an insight particularly relevant in a phenomenon as intricate as chronic pain. The increase in functional disability appears to depend on the inclusion and interaction of more psychosocial, clinical and impaired pain modulation components, suggesting a cumulative effect. The results presented here clearly demonstrate that factors related to the disease and protective mechanisms (pain modulation) interact inversely to establish greater pain and disability, contributing to a more complex patient profile. In this context, we emphasise and encourage the importance of patient phenotyping studies to enhance understanding of the interaction between disease‐related factors and the degree of functional disability.

This study has certain limitations that should be acknowledged to ensure cautious interpretation of the findings. First, the cross‐sectional nature of the design precludes any inference of temporal or causal relationships between the variables analysed. Additionally, it is a secondary analysis of cross‐sectional data, and a control group without TMD was not included due to the lack of sufficient clinical data related to pain duration, frequency and comorbidities. Despite these limitations, only validated questionnaires were used for data collection, and an experienced professional conducted the clinical examination and performed the QST and CPM tests. We propose that future longitudinal investigations reassess whether these responses remain consistent when patients are evaluated over time.

## 5 Conclusion

The results identified five main components that influence the severity of disability associated with chronic pain. The clinical pain and psychosocial components were progressively more prominent in the groups with greater disability, reflecting an accumulation of clinical and psychosocial factors in individuals with more pronounced functional impairment. Additionally, the pain modulation component was inversely associated with the highest levels of functional disability, indicating a lower likelihood of belonging to these groups among individuals with better pain modulation capacity. These findings clarify that disability related to chronic TMD results from a cumulative and interactive load of clinical and psychosocial factors, as well as mechanisms related to endogenous pain inhibition.

## Author Contributions

All authors contributed to the study conception and design. Material preparation, data collection and analysis were performed by Rafaela Stocker Salbego, Matheus Herreira‐Ferreira, Beatriz Amaral de Lima Netto, Tiffany Karoline Barroso Santos, Yuri Martins Costa, Paulo César Rodrigues Conti and Leonardo Rigoldi Bonjardim. The first draft of the manuscript was written by Rafaela Stocker Salbego and all authors commented on previous versions of the manuscript. All authors read and approved the final manuscript.

## Conflicts of Interest

The authors declare no conflicts of interest.

## Peer Review

The peer review history for this article is available at https://www.webofscience.com/api/gateway/wos/peer‐review/10.1111/joor.70008.

## Data Availability

The datasets generated and/or analysed during the current study are available from the corresponding author upon reasonable request.
